# Negotiating an Illicit Economy in the Time of COVID-19: Selling and Buying Dilemmas in the Lives of People Who Use Drugs in Scotland

**DOI:** 10.1177/00914509221122704

**Published:** 2022-09-08

**Authors:** Angus Bancroft, Tessa Parkes, Idil Galip, Catriona Matheson, Emma Crawshaw, Vicki Craik, Joshua Dumbrell, Joe Schofield

**Affiliations:** 1 The University of Edinburgh, Edinburgh, United Kingdom; 2 University of Stirling, Stirling, United Kingdom; 3 Crew, Edinburgh, United Kingdom

**Keywords:** COVID-19, moral economy, Scotland, people who use drugs

## Abstract

The impact of COVID-19 itself and societal responses to it have affected people who use drugs and the illicit drug economy. This paper is part of a project investigating the health impacts of COVID-19 related control measures on people who use drugs in Scotland. It examines their roles and decisions as economically situated actors. It does this within a moral economy perspective that places economic decisions and calculations within a context of the network of social obligations and moral decisions. The paper uses a mixed methods approach, reporting on a drug trend survey and in-depth interviews with people who use drugs. It finds they were affected by restrictions in the drug consumption context and changes in the supply context, both in terms of what was supplied and changes in the relationship between sellers and buyers. Face to face selling became more fraught. Participants in more economically precarious circumstances were faced with dilemmas about whether to move into drug selling. The double impact of loss of income and reduced access to support networks were particularly difficult for them. Despite the perception that the pandemic had increased the power of sellers in relation to their customers, many full-time sellers were reported to be keeping their prices stable in order to maintain their relationships with customers, instead extending credit or adulterating their products. The effect of spatial controls on movement during the pandemic also meant that the digital divide became more apparent. People with good access to digital markets and easy drug delivery through apps were in a better position to manage disruption to drug sales contexts. We make recommendations in relation to how policy can respond to the interests of people who use drugs in a pandemic.

## Introduction

During COVID-19 times, as with other epidemics, people who use drugs may be more vulnerable both to the disease itself and to the control measures introduced to manage it that may further disrupt their lives (Chang et al., 2020). The focus of this paper is the way in which changes in the illicit drug market and distribution systems have shifted use and selling contexts and decisions. The paper is part of the project “Understanding the health impacts of the COVID-19 response on people who use drugs in Scotland: Implications for COVID-19 infection/transmission and impacts on harm reduction, treatment and recovery” (Matheson et al., 2020). It investigated the long-term health impacts of COVID-19 control measures on people who use drugs. It also examined changes in drug distribution and consumption, access and availability of harm reduction and other health services, and the provision of addiction treatment services. In this paper we focus on how sellers and buyers of illicit drugs respond to these control measures and disruptions and to the dilemmas created by them. We examine how their roles as economic actors and as people who use drugs are entangled and present complex dilemmas, such as whether and on what terms to take up drug selling, and how to manage changes in income, and deal with unpredictable drug quality and availability in a scarce resource environment.

Illicit drug markets have historically been subject to various shocks and challenges. There are those which are typical of any economy: changes in consumer preferences, costs of production, labor and supply problems and innovations (Ciccarone, 2019; Moyle et al., 2019). However, in addition they also face hostile disruption from law enforcement (Nelson, 2018), and potentially violent competition from rival groups (Bakken et al., 2018). They are bound up with the global political economy, though in unusual ways, operating sometimes through influential economic and political entities (Ferreira, 2019). Generally they exhibit resilience to most shocks, showing a high degree of cooperation, networked intelligence, and responsiveness to threats (Golz & D’Amico, 2018). They usually function cooperatively, but selective violence is used during times of competition and disruption (Friman, 2009). COVID-19 was expected to be an unprecedented challenge due to the literal and figuratively global nature of its impact (Zolopa et al., 2021). Lockdowns and other disruptions affected multiple systems simultaneously, though not to the same degree everywhere. Disrupted global labor and distribution patterns had localizing effects, particularly increasing incidence of failure in cross border drug transactions recorded in cryptomarkets (Bergeron et al., 2022). Established supply and consumption patterns were destabilized (Ali et al., 2021). A minority of users reported reduced drug quality (Scherbaum et al., 2021) and greater adulteration as sellers sought to manage their response (Voce et al., 2020). Some had fewer contacts with sellers as a result of lockdown (Otiashvili et al., 2022). This paper argues that these effects are distributed unevenly depending on the varied economic and social embeddedness of users and sellers. The context is one in which integrated, densely connected local working class communities have been hollowed out by de-industrialization, so that the basis for illicit organization and action is through loose, temporary groupings that seek to dominate market segments rather than control territories (Hobbs, 1998). They are flexible in response to challenges and adapted to changes in the drug market (Lankenau, 2013) and driving changes in local markets in response to changes in global supply conditions.

Due to the precarious and informally governed nature of the illicit drug economy, people who use drugs in marginalized social and economic circumstances maintain a moral economy through a complex set of reciprocal obligations and responsibilities (Bourgois, 1998; Wakeman, 2016). The moral economy combines a range of actors and institutions. It includes people who use drugs, their friends and families, sellers, treatment centers and support services, police and social work. Each has different power and influence in the moral economy. They produce a complex network of obligation, surveillance, dependence, punishment and provision.

The illicit moral economy manages a set of instrumental requirements: transactions have to be reliable without the benefit of outside agencies to appeal to. Buyer and seller need to coordinate and reach agreement on pricing in a context where there is a significant power disparity between them (Beckert & Wehinger, 2012). Despite the absence of enforceable agreements, exchanges are mostly carried off without threats or actual violence (Reuter, 2009). The moral economy limits what otherwise might be predatory and violent interactions (Moyle & Coomber, 2017). It binds buyers and sellers into norms of reciprocity and sharing, distributing resources and helping them limit withdrawal and also avoid certain harms such as overdose. It works both through calculated instrumental reciprocity and emotional bonds of care and mutual support (Harris & Rhodes, 2013; Wakeman, 2016). However, there are circumstances where it can be put under severe strain such as situations where the market is severely disrupted. Its working can also threaten harmful consequences, for instance in generating obligations that can represent dilemmas for people who want to withdraw from selling or change their drug use.

In Scotland, as elsewhere (Mathew et al., 2021), problem drug and alcohol use are closely intertwined with a context of multiple, historic deprivation. 13.5% of adults in Scotland used drugs during 2018/19, an increase over the past decade (National Statistics, 2021). 57,300 people in Scotland are categorized as problem drug users, which is around 1.62% of the population (Information Services Division, 2019). People who use drugs between 35 and 54 years of age are particularly at risk and, given the nature of their vulnerabilities, we expected COVID-19 related disruptions to drug supply to be especially dangerous for this group. In 2020, 1,339 drug related deaths were recorded: a historic high. There are also prevalent comorbidities. Dependent users of alcohol and other drugs are at risk of severe responses to COVID-19 due to co-occurring risk factors such as smoking, comorbidities such as cardiac and respiratory problems (Benzano et al., 2021), and worsening psychological problems (Zvolensky et al., 2020).

At the start of lockdown several hypotheses were proposed by the research team that: people who use drugs would stockpile in response to the risk of unreliable supply; risky behavior would increase as people who use drugs turned to unreliable sources or unfamiliar drugs; and consumption patterns would change as the result of changes in lockdown lifestyles. Various mechanisms affect consumption during times of economic upheaval (Nagelhout et al., 2017). People who use drugs may have fewer resources to buy drugs and may reduce use, move to cheaper products, or to alcohol. The quality of the drugs being sold may suffer and so encourage people who use drugs to substitute a drug familiar to them with another which is an unknown quantity or which carries greater risk. Countervailing tendencies, such as greater stress, disruption to social networks, and anomie, can also fuel demand for drugs and alcohol during a recession (Nagelhout et al., 2017). The focus of this research was on changes to the everyday nature of selling and buying practice in this context. We wanted to see how a group of people who use drugs who were often excluded from the digital society responded to the pressures of the pandemic, how they made assessments about drug potency and dosage in uncertain times, and the ways in which it presented a further challenge in maintaining the illicit economy during extensive disruption.

## Method

The research design consisted of two elements, a survey which provided information on changes in the population drug use context, and a qualitative interview study to capture the experiences of people who use drugs during lockdown. We wanted to understand the context of changing patterns of drug supply and use, and how people who use drugs responded to those challenges alongside the many other problems lockdown presented for them, such as potentially reduced income. We did not limit the study to specific groups of people who use drugs.

The methods combined data from a rapid response survey and qualitative interviews. Survey data was provided by our project partner, Crew, the Scottish harm reduction and outreach charity which surveyed people who use drugs and drug services throughout the pandemic. The “COVID-19 and drug markets” survey was conducted online. The survey was conducted in two phases, April and May 2020. There were 327 responses to the April survey and 50 to the May survey. The survey aim was to understand the impact on people who buy, sell and consume drugs on the impact of COVID-19 and associated restrictions. Responses were requested from people who take drugs and those who work with them. Survey questions covered: changes in the frequency and quantity of drug use as a result of COVID-19; types of drugs taken; changes in the mode of drug purchase and the drug market in general; anxieties due to changes in the way drugs are bought, sold and taken; effects such as withdrawal; difficulty accessing prescriptions and support. Open text comment was invited on the reasons for changes, and other experiences. Further demographic data was not collected by the survey. Descriptive statistics were used from the survey to understand the impact of the pandemic on the drug using population overall. Ethical approval was granted by the University of Stirling’s General University Ethics Panel (GUEP, 916) and The Salvation Army.

Twenty-nine interviews were conducted between July and October 2020 with participants who were over 18 years of age and who identified as using street drugs and/or receiving treatment for a substance use problem. This included 13 women (33–44 years) and 16 men (28–56 years). 16 were recruited via a homeless hostel, two from a recovery community, eight from a stabilization service, and three from another support service so we can infer that at least 16 were homeless at the time of interview. The sampling was designed to capture a range of experiences according to age, gender, and treatment and drug use status.

The settings included a homelessness residential service (hostel/shelter), a stabilization and housing service, a harm reduction service, and a peer-led recovery community. Participants were aged 18 or over and self-identified as currently using street drugs and/or receiving treatment for a substance use problem. Clients were recruited via service managers who were asked to discuss the project with eligible clients, seeking to include a balance of genders and people at different stages in relation to their drug use (currently using drugs and/or in treatment and recovery). Clients who wished to participate could either contact the research team directly by telephone or email or ask a member of service staff to pass on their details. No information was available on the number or characteristics of PWUD who were approached but declined to participate.

The interview topic guide covered: changes to drug use since the start of lockdown, access to and use of drug-related services (harm reduction, opiate replacement therapy (ORT) and recovery services), other health and support services, and impacts on physical and mental health. Interviews were conducted in person or via telephone. JD is a community researcher and a homeless service worker with lived experience of problem drug use and homelessness and being personally known to some participants assisted in recruitment. Written consent was obtained when conducting face to face interviews and oral consent for telephone interviews. Interviews were audio recorded, transcribed in full, and analyzed thematically.

Descriptive statistics were used from the survey to understand the impact of the pandemic on the drug using population overall. Interviews focused on the impact of the pandemic on the lives of people who use drugs. Interview data were analyzed using the Framework Method (Ritchie et al., 2003) in NVivo 12 (QSR International Pty Ltd., 2020). An inductive approach to analysis was used in which a coding framework was iteratively developed using themes emerging from the data. JD read four transcripts in full and these were coded line-by-line to identify emerging themes which were then reviewed by him. The team reviewed the coding framework. This framework was then applied to the full dataset. New categories were added if they emerged. Seven interrelated high-level themes were identified, four of which were drawn on for this paper.

## Findings

### Supply and Consumption Contexts and Risk Responses

Changes in the drug market had a different salience depending on how users were positioned in relation to the drug market. Survey and interview data both reflected changes in drug supply, demand and drug consumption contexts. The most striking difference was the way that these were experienced (Table 1). Survey respondents experienced changes in drug use opportunities driven by the COVID lockdown, leading to reduced demand for “club drugs” (drugs traditionally taken in recreational settings such as clubs and festivals) such as MDMA/ecstasy and amphetamine. Opportunities to use these drugs in recreational settings were heavily restricted, and many respondents reported loss of income, boredom and anxiety. A majority (57%) increased the frequency of their drug use and also (60%) switched drug types due to availability and preference.

**Table 1.** Response Summaries From Scotland-Based Survey Respondents.

	Yes	No	Other
Noticed changes to drug supply since COVID reached Europe	63.6%	30.9%	5.8%
Changes to the way drugs bought sold or taken caused any worry or feelings of anxiety	73.7%	26.3%	—
Experienced unintended withdrawal symptoms due to COVID-19	42.6%	57.4%	—
Difficulty in getting support related to drug use due to COVID-19	63.3%	36.7%	—

Vulnerabilities among people dependent on drugs or who relied on treatment were being exacerbated. Open text survey responses reported ongoing mental health problems, and increased concerns about domestic violence/coercive control. 73% said that existing or expected changes in drug supply had caused them anxiety as existing opportunities to purchase and use had become limited. Some were concerned about their ability to maintain drug use, being pushed into higher risk use and maintaining access to drug support services. Interviews confirmed much of this. Interviewees who increased use reported boredom, stress, insomnia and other effects of disrupted life patterns. Those who reduced their use noted a significant reduction in income and buying power, as well as the greater expense of certain drugs. Expending greater effort to find drugs, or find the money for them, added to anxiety and stress.

There was a general sense of pre-pandemic trends continuing. Heroin potency had been recorded as falling several years prior to the pandemic and this trend continued and was heightened during lockdown:

Heroin’s always been very, very weak, for I don’t know, about 20 years. It’s probably been pretty weak, up in my bit, probably since, no maybe about 15 year probably. Just not been that very good, so that’s how you’ve got to Valium on top of it, you know what I mean? (Z6, unknown)

Crack on the other hand was reported in interviews as notably weaker, and much more adulterated due to the lockdown. These changes in supply meant decisions had to be made about consumption. There were those who made the decision to balance potency, moving from weaker crack to stronger benzodiazepine. Potency was not a straightforward benefit. Unusually strong batches of heroin would lead to overdose as people had become used to a very weak product. C3 described their mistrust of benzodiazepines strength:

I don’t really trust [benzos], because you don’t know what’s in these ones these days. If I could get pharmaceutical ones, like the ones, the ones out the chemist, and you see the wee prescription bag, I would buy some of them. But other than that, I wouldn’t buy they ones, they white roses and that, because you don’t know what’s in them, and you wake up on the street, phew, you don’t know where you’ve been, who you’ve been with. (C3, M, 44)

Interviewees had to deal with several uncertainties, knowing what drugs had changed, and how to rely on good harm reduction practice in the light of much greater uncertainty about drug content. Survey respondents noted shortages, reduced quality and reduced variety, and decreased interaction with sellers. For example, sellers no longer messaged with special offers and new products.

Policing also affected people’s ability to generate income, for example through begging:

During lockdown it was hard, really hard, because any time you went to sit down somewhere you were getting moved. So you could sit down for 20 minutes somewhere, and you were getting moved, anywhere in the city, anywhere, which was hard. (C5, Female, 33)

There were those who were impacted positively by assertive outreach to adopt rapid prescribing of ORT, or who changed from using multiple times a day to gaining stability and using only on payday. They appeared to view the lockdown as an opportunity to reduce drug use, and/or to use “less harmful” substances such as cannabis or alcohol:

I know it was a bad thing that happened, but it really benefited me, in, in the long run. Gave me the boot up the backside I needed to get, pushed myself to get the prescription, I was trying to go on a prescription anyway, and when I was on the dihydrocodeine for about a week or two, and then it was…methadone. (C3, Male, 44 years)

As well as changes in drug use patterns, interviewees described how their risk calculus had changed. Several reported a change to the type of drugs they were taking, for example starting to use crack cocaine, despite issues of reliability outlined below. A few reported they had moved to riskier routes of administration, such as injecting cocaine. They linked that change to availability with easier access to those drugs and opportunities to inject.

It’s like putting it in your face, and you think if I’ve got a bit of kit [heroin] in my pocket, I was just going to burn it, the next minute somebody’s offering me needles, oh fuck it, I’ll take them, I shouldn’t have done that, what happens if I OD or anything, I’m no wanting to do that again, but now I’ve got a drawer full of needles. (C2, M, 31)

As with changes in drug use types, the restricted set of contexts users can buy and consume drugs altered drug consumption practice. A group of interviewees in one site was reported to have shifted to injecting cocaine use, something that demonstrates how risky practices can develop as localized responses to changes in supply. A puzzle that became apparent during the study was how users were positioning themselves in relation to these changes which we came to understand in terms of how the drug market was mediated technologically and interpersonally.

### Users’ Embedding and Disembedding in the Drug Market

The drug market works specifically as a set of techno-social systems in which drug users are embedded differentially depending on the factors like their socio-economic and cultural position. Here we are setting out the market as a set of relationships, and examine how mediation of these relationships has changed due to COVID. The ability to continue to obtain drugs depended on income, opportunity to buy, and place in the existing drug supply network. This combination of a specific technical-social environment was illustrated by one user-sellers had an established relationship with his supplier:

It wasn’t that, that hard to get them still. Just contacting the person, because he’s in prison doing a very long prison sentence, that was, there was a couple of times that was a wee bit hard, because they stopped doing visits in prison, so I got over any hurdles with the visits or the contact, the prison gave out mobile phones, so it meant he had two mobile phones and, so I could phone him any time. (T7, M, 32)

Their relationship continued while the supplier was in prison. The prison gave out mobile phones during lockdown and this helped the communication continue. This interviewee’s selling later came to a halt when his phones and cash were seized by the police. Policing can interfere with drug transactions in unexpected ways and this incident highlights how the effect of policing varies significantly depending on the social position of people who use drugs and drug sellers.

Positioning in relation to the drug economy was a combination of social network position, cultural knowledge and felt obligation. This interviewee described a reluctant process of being drawn into a supporting role for other drug sellers:

Somebody phoned me the other day, “have you seen so and so,” “aye, he was there,” “go and find him for us, and when you get him, stay with him till he phones me, and I’ll get him to give you a rock for it?” and I’m like, “I’m no wanting a rock for it,” you know? But that’s, you know, and it’s no that, after that, and he was like, ‘oh, and another thing, when you get your phone, send me your number, I’ve got a couple of things here for you, I didn’t ask for anything, you know what I mean? I get offered it. (C8, M, 38)

C8 wanted to avoid the stress of selling and becoming responsible for other people’s drug use but found a strong pull as others made use of him to monitor their customers. His experience is an instance of refusing easy money or access to drugs because of the obligations he would have to take on if he did accept payment. Interviewees like C8 above recognized the existence of a moral economy that could involve reciprocal obligations. The below interview is with a person who used and sold diazepam and heroin. T7 highlighted the emotional costs of involvement in selling:

I was stressed a lot, but that’s, I think a lot of that was due to, I was relied on, see when you, when you sell heroin, you’re, you are relied on very much. I mean it doesn’t matter if you go to bed at 4 o’clock in the morning, you get up at 9 because there’s people relying on you for their habit. (T7, M, 32)

According to interviewees, selling practices were adapted to the lockdown. As these accounts show, people were embedded in the drug market through interdependent networks. Some of these obligations could be onerous. Sellers stretched their customers’ time, keeping them waiting when transaction times had been arranged.

Interactions with sellers had changed. A common experience among participants was being “bumped” (being sold something other than what was expected or agreed). They reported having difficulty challenging being bumped due to the hurriedness of transactions. Those who were involved in selling described bumping others and also as a result living heightened anxiety, paranoia and credible threats of violence. This change in the emotional texture of drug transactions went along with reduced information along the supply chain:

It was harder to be honest, a lot of the dealers couldn’t get a hold of supplies, which meant we were having to go further afield to try and find the drugs, which means we don’t know the people that we’re getting them from, we don’t know what they’re cut with, and the prices go up as well, yeah. (C5, F, 33)

Individuals who engaged in selling within our sample did so as a means to support their own use. The low cost of street benzos meant that it was common for individuals not historically engaged in selling drugs, to buy large batches and to distribute enough to cover costs and personal use. 4 or 5 shared such experiences.

For interviewees who engaged in selling, their position and reputation were key to maintaining their position. Reputation was maintained through conducting repeated transactions reliably.

No, no, they want me to deal for them, because they know that I’ll no get taxed. They know that their money will be there when it’s meant to be there, and that, you know, because if I’m, like before when I used to sell drugs and that, when it comes to drugs, I don’t muck about, because drugs can get you murdered. Even a tenner can get you, well I’ve seen guys get stabbed to bits over a fiver, you know what I mean? Or even one Xanax, so it went, it tends to be drugs and things like that. I don’t muck about, you know, I try and be as transparent and as straight as I can. (C8, M, 38)

People positioned higher up in the supply chain were also affected by COVID restrictions. Being well connected was reported to place additional demands on participants. For example, C8 was subject to continued demands from “higher level” sellers, and people who knew him as someone who “knew the right people” during lockdown, when other sources of drugs dried up. The impact of lockdown was to disrupt reciprocity and resource pooling. Opportunities to pool resources were closed down as begging became impossible.

My mate was a drinker, and the rest of them were like addicts, about 5, 6 of them. Now they all sit at different bits of the, you know, in the town, begging, and then we’d all phone one another, right, you ready to go and score, we’d to go score together. That was us, go and score, back up to the town, make some more money. Crazy. (C3, M, 44)

In this instance sociability and togetherness were halted. People found themselves atomized and disconnected from this source of resource sharing, this element of the moral economy now disrupted. Disruption to resources, and changes in personnel signaled the emergence of a more exploitative context. There was much more pressure from both ends: limited income and also greater competition in terms of people willing to and needing to take up roles as sellers.

Out right now and trying to sell, because they’ll know that the homeless will be looking for something, so they’ll be trying to mix something together to try and get the money out of us, do you know what I mean? (C5, F, 33)

New faces had come onto the scene and there was a sense that the moral economy had shifted to one that was more instrumental and exploitative:

Just more, that’s [name] trying to get a hold of me, they’re just harder, crueller, trying to grab as much money as they can get, and get as much as they get, and consuming far too much as well. (C6, M, 43)

Conflict emerged as people were sold very low quality products, such as paracetamol substituted for crack. Sellers made use of their greater power:

They play God with their stuff, because they know the situation, so it’s like they, they like to play God, they choose and pick who they want to give to. (T3, M, 45)

One interviewee stated that COVID-19 had “actually produced more dealers,” and many others share the perception that there are new and younger runners (low level distributors) and sellers on the street since the beginning of the pandemic. Participants also stated that sellers have “been struggling,” most likely due to increased competition and decreased demand. This increased competition within the market was reported to have led to sellers acting more aggressively in a bid to protect their positions within the marketplace. Participants who discussed that they had been feeling more mistreated by their sellers than usual linked this change in behavior to the ways that sellers exploited the pandemic conditions and consequent anxieties that people who use drugs experienced regarding supply shortages or drops in quality.

On the other hand, despite the greater opportunities presented for price gouging, many sellers did not seem to use the situation to increase prices. They instead reduced the potency of the drugs they sold or used other strategies such as extending credit. Expanding use of credit was used to maintain a buyer base and may also have smoothed out the immediate impact of price rises. We see here how the moral economy involves both pragmatic adjustments and a sense of a forward trajectory with sellers who would try and maintain a basic selling relationship with their customers. Incidents of violence were not reported in our interviews, however “bumping” was a problem. Sellers were not wholly concerned with exploiting the situation. Extending credit to their customer base keeps their market alive and also generates debt which needs to be paid. The story overall was one of localized, sometimes subtle shifts in power and selling practice.

### The Changing Balance of Digital and Face to Face Relationships in Drug Transactions

Our survey data showed that 33% more people reported using the internet (including the darknet) and social media to purchase drugs during COVID. The lockdown meant empty streets and established sellers were making less use of street pitches to deal, instead moving to point to point selling where they arranged to meet customers at specific times and places. As other sellers’ supplies were restricted, participants had to venture further afield to buy from people unknown to them. There was also greater adulteration of supply, making them uncertain of the quality of drugs that they were buying.

There were also changes in risk patterns in drug selling, due to concerns about contracting COVID-19. For instance, sellers were shifting from selling on the street to selling from their cars. When sellers change their terms of business it is presented as a “take it or leave it” proposition. Interviewees highlighted that they had little power over how and when these changes occur, something that aids in creating a further power imbalance between drug sellers and people who use drugs. The pandemic sharpened the distinction between people who were able to use digital drug purchase means on their terms, acting as “consumers.” For those who do not have much control over the relationship they become something more like “clients.”

Despite these new challenges, interviewees expressed reluctance to switch to app based or internet based supply. Digital devices were often seen as a source of risk and vulnerability, to financial scams, for instance.

I don’t know, I know there’s a lot more of it going about, than what there ever used to be, cause even on Snapchat you’re able to get these people that are selling stuff now, all over [town], which is quite scary…I think Facebook used to…is bad for that, obviously folk get to you easily. But Snapchat, I think because when they send you a message, or a picture of something, it’s gone, it’s deleted after 10 seconds or something. (T5, F, 47)

Mainly they relied on personal contacts and the mobile phone network. That increased the time they had to spend finding a dealer and waiting on them:

It was, you’ve got to get one of your pals to, I couldn’t talk much…I had to get somebody else, I’ve had to phone a few times eh, because like with the other people, it was normally, we’ll put this, well a couple of them has been put out now, so […] Prescribed, there wasn’t, I wouldn’t say it was any harder like, it was longer, like the phone calls and stuff like that, and longer we, with having to wait on people, because they’re too dodgy with hanging about eh. (T2, F, 44)

In contrast to more affluent people who are able to set the terms of their engagement with the illicit market and have sellers come to them, many interviewees reported the opposite experience: a context of being made to wait, being cancelled on, or otherwise having to go to greater effort to secure sometimes limited supplies. For those in the broader drug-using population responding to the survey who were more digitally connected, smartphones and apps worked to mitigate lockdown, while for interviewees digital systems became another vector of perceived exploitation.

## Discussion

The findings are gathered into the following points: changes in the supply chain, existing inequalities and power differentials, positioning in the moral economy, and digital connection and disconnection. We observed a set of changes the material-technical context of the moral economy of PWUD.

While most of UK society was affected by the lockdown and many people experienced isolation and other negative effects this change fell differently depending on PWUD’s social connectivity, and position in relation to digital society. The illicit market manifests separately depending on the cultural and material resources available to users. How the market is mediated has changed during the pandemic, affecting face to face relationships and users’ relationship to the technological mediation of the market. Our findings illustrate how agency is redistributed in relation to both drug use contexts and the supply chain. Overall some users—those who were well situated—remained embedded in the drug market with minimal disruption, some were disembedded and had to reconfigure their relationships with sellers, some were disembedded and sought treatment or reduced use as a result. That middle group was most at risk of problems as a result of the shifting moral economy. These included loss of reciprocal relationships, greater risk of exploitation in drug buying/selling, and resultant personal stress and mental health difficulties.

Digital technologies can be a vital link for PWUD. Some do not own devices or are wholly digitally excluded. Of those that do they tend to connect to the internet through smartphones, with a minority owning laptops and few owning desktops (Matheson et al., 2022). The nature of access to online services and information is shaped by that. Being digitally adept allowed some users to substitute existing supply lines but overall was another factor which shifts and mediates relationships between offline and online. Digital access is best viewed as one part of a material relationship. People who sell drugs who operate from a house, or another space they control, who have a stable internet connection, their own fixed phone line, or access to darknet markets and other online sources, were able to maintain a more stable position in the supply chain. Those who are already the least powerful in the drug market were finding it hard, particularly those who were also selling or considering starting to sell drugs as other sources of income were drying up. At the same time, competition between people who sell drugs was reported as increasing. One response was to limit their interaction with the monetized illicit economy and avoid taking up all earning opportunities available to them. A role they were wary of was the dual user-sellers role which rendered them vulnerable in several ways. The digital therefore functioned to redistribute vulnerability and changed participants’ risk calculus about the terms they were able to engage in the drug market on. Their sense of their own trajectory mattered, with those who had been concerned about harmful or addictive use sometimes managing to change their use pattern.

Digital inequalities arise from the inequalities in the distribution of economic, cultural and social resources needed to make use of digital technologies. Having access to a smartphone does not always mean that the owner of the smartphone has the digital literacy to participate fully in a digital social life. These digital inequalities often intersect with off-line inequalities such as class, race, gender, and geographical origin. While some have the means and opportunities to seek out and maintain social and economic interactions online, more vulnerable people might not be able to make full use of what digital technologies have to offer (Robinson et al., 2015). This becomes particularly alarming as digital communication and spaces are increasingly becoming necessities rather than amenities (Beaunoyer et al., 2020). Within the context of COVID-19, people who use drugs who have the digital literacy to be able to observe, access and use online illicit markets to buy drugs can avoid face-to-face interactions with sellers and potential exposure to COVID-19, among other risks. However, this does not mean that risks, moral obligations and reciprocity do not play a part within digital transactions. Offline and online risks often intersect, as the boundary between them gets increasingly hazier, with people who use drugs adapting to different risks that online drug buying contexts present, for instance cultivating online reputation and trust, purchasing drugs across borders, receiving shipments to home addresses, or online scams (Masson & Bancroft, 2018).

Following on from that, we should understand observed changes in price and purity of illicit drugs in terms of PWUD’s capacity to manage their relationship to the drug market, the moral/personal obligations they feel toward others and that others have toward them. Understanding pragmatic decisions about what to do in response to short supply or rising prices was vital as these decisions affect participants’ health and the risks they are willing to take. Beyond that it was also significant for their own relationships and sense of who they were as social individuals, and the choices they made about drug consumption and treatment. These knock-on effects could prove significant in the lives of people who use drugs, especially those who occupy particularly risky positions like user-sellers roles (Moyle & Coomber, 2015). Some found it most protective when they could start extricating themselves from it, using the pandemic as a reason to move into ORT support or treatment or limit their involvement in selling.

Therefore we need to contextualize conclusions such as drug supply being more resilient than expected. Apparent resilience disguises the labor needed to maintain the drug market and the shifting relationships within it. It was drug buyers and user-sellers who were presented with the greatest challenges in adapting purchase and consumption practices. For the sample, the impact of the lockdown and disruption to global trade did not lead to the expected across-the-board increase in drug prices and drop in quality. There was lower quality and lower choice and variety, and sellers faced challenges as their user base had been reduced due to lower consumption and lockdown restrictions. Where people have fewer resources to fall back on, then they are more vulnerable to victimization. The market changes due to COVID re-emphasize existing inequalities, much as they do in the licit economy. Positioning in the moral economy was relevant to how participants experienced changes in the market. Drug markets are embedded in a web of reciprocal obligations, affective relationships, financial and moral debts and other ties which bind buyers and sellers together. These relations distribute risk and opportunity, reward some and disadvantage others. That meant we could not view the disruption to the market purely in terms of its practical effects but also must consider it in terms of the social networks people embed in, and recognize that drug sellers can be part of care networks (Kolla & Strike, 2020).

For the most vulnerable people who use drugs the moral economy is closely tied into their support network. Loss of income and reduced access to the support networks are a double whammy for them. Some reported sellers behaving more aggressively as a result of greater competition and lowered demand. The close relationship with the moral economy means that less affluent people have reduced room for maneuver when experiencing disruptions like that and limited resources to fall back on. The pandemic showed the limits of the cash and street nexus. Interviewees highlighted the limits that lockdown placed on the street as a place to earn money and conduct deals. According to our data there was a decline of face-to-face drug purchasing during the pandemic and a further rise in the popularity of social media apps for obtaining drugs. It is likely that these changes further marginalize the moral economy of already vulnerable people who use drugs.

We make the following recommendations:

1. The interests and wellbeing of people who use drugs who are living in vulnerable circumstances should be taken into account when designing pandemic response policies. In particular, there are unanticipated knock on effects on their ability to maintain their income to consider.
2. Some assumptions have been made about how different digital platforms and modes intersect in the lives of vulnerable people who use drugs. Being connected to online communities and cultivating online networks of care can be key in managing isolation, or even unwanted reciprocal obligations faced in offline spaces, such as being pressured into taking on a user-seller role. Online communities, especially those focused on harm reduction, can offer alternative networks of care for people who use drugs. Access to these networks can be challenging to develop for more vulnerable people. Where more affluent people can fall back on home networks and deliveries, those in more precarious and resource scarce circumstances are affected by library and service closures that make access to Wi-Fi more difficult. Further developments could support marginalized people who use drugs in their use of online harm reduction forums.

### Strengths and Limitations

We decided to analyze the impact of market changes on people who use drugs as socially situated market participants, rather than as purely economic actors. Using the different data collection methods allowed us to view the market in terms of a range of participants who occupied different roles and positions within it. The interview data meant we were able to understand how participants positioned themselves and approached the drug market in the context of their own resources and experience. Using these methods allowed us to understand the balance of their agency and structural and economic forces affecting their lives and choices.

#### Limitations

- Interviews were one-offs. The pandemic has long term consequences and follow up, longer term interviews would allow researchers to scope the continuing impact on people’s positioning within drug markets.
- Interviews did not sample from the National Health Service’s drug services and worked with the third sector only, and we did not access those who were not in touch with any services. People who rely on “invisible services” such as peer self-care and informal online support may have distinct ways of adapting to the challenges we identify.
- The survey was a non-probability sample and demographic data collected by the survey was limited and so information about respondents’ social status and typical drug use had to be inferred from free text comments. This limits our ability to generalize about the relationships between social class/status, gender, sexuality, ethnicity, urban/rural geography and drug market position.
- Respondents and participants could be sampled/typified by their mode of drug acquisition, allowing us to investigate how COVID related disruptions affect the balance between social and commercial supply relationships.

## Conclusion

The study aimed to explore the varied health impacts of the pandemic and associated policies on people who use drugs in Scotland. This paper sought to understand how market-specific changes had affected their experiences and the choices they made. It used a combination of a drug trend survey and interviews with people who use drugs. The moral economy perspective was used in order to situate people within the social context in which they took decisions to buy or not to buy, to change their drug use patterns and habits, to deal or not to deal, and allowed us to understand the price setting decisions of sellers. In each case we could see how the decisions were taken within a complex network of opportunity and obligation. That could include the opportunity to stop using altogether, to limit use, to change use patterns or to change one’s own position in the illicit drug economy.
